# Marine omega-3 polyunsaturated fatty acids induce sex-specific changes in reinforcer-controlled behaviour and neurotransmitter metabolism in a spontaneously hypertensive rat model of ADHD

**DOI:** 10.1186/1744-9081-8-56

**Published:** 2012-12-10

**Authors:** Kine S Dervola, Bjørg Å Roberg, Grete Wøien, Inger Lise Bogen, Torbjørn H Sandvik, Terje Sagvolden, Christian A Drevon, Espen Borgå Johansen, Sven Ivar Walaas

**Affiliations:** 1 Department of Biochemistry, Institute of Basic Medical Science, Faculty of Medicine, University of Oslo, Oslo, Norway; 2 Department of Physiology, Institute of Basic Medical Science, Faculty of Medicine, University of Oslo, Oslo, Norway; 3 Department of Nutrition, Institute of Basic Medical Science, Faculty of Medicine, University of Oslo, Oslo, Norway; 4 Institute of Psychology, University of Oslo, Oslo, Norway; 5 Oslo and Akershus University College of Applied Sciences, Oslo, Norway

**Keywords:** Omega-3, ADHD, Behaviour, Dopamine, Serotonin, Glutamate, Neostriatum

## Abstract

**Background:**

Previous reports suggest that omega-3 (n-3) polyunsaturated fatty acids (PUFA) supplements may reduce ADHD-like behaviour. Our aim was to investigate potential effects of n-3 PUFA supplementation in an animal model of ADHD.

**Methods:**

We used spontaneously hypertensive rats (SHR). SHR dams were given n-3 PUFA (EPA and DHA)-enriched feed (n-6/n-3 of 1:2.7) during pregnancy, with their offspring continuing on this diet until sacrificed. The SHR controls and Wistar Kyoto (WKY) control rats were given control-feed (n-6/n-3 of 7:1). During postnatal days (PND) 25–50, offspring were tested for reinforcement-dependent attention, impulsivity and hyperactivity as well as spontaneous locomotion. The animals were then sacrificed at PND 55–60 and their neostriata were analysed for monoamine and amino acid neurotransmitters with high performance liquid chromatography.

**Results:**

n-3 PUFA supplementation significantly enhanced reinforcement-controlled attention and reduced lever-directed hyperactivity and impulsiveness in SHR males whereas the opposite or no effects were observed in females. Analysis of neostriata from the same animals showed significantly enhanced dopamine and serotonin turnover ratios in the male SHRs, whereas female SHRs showed no change, except for an intermediate increase in serotonin catabolism. In contrast, both male and female SHRs showed n-3 PUFA-induced reduction in non-reinforced spontaneous locomotion, and sex-independent changes in glycine levels and glutamate turnover.

**Conclusions:**

Feeding n-3 PUFAs to the ADHD model rats induced sex-specific changes in reinforcement-motivated behaviour and a sex-independent change in non-reinforcement-associated behaviour, which correlated with changes in presynaptic striatal monoamine and amino acid signalling, respectively. Thus, dietary n-3 PUFAs may partly ameliorate ADHD-like behaviour by reinforcement-induced mechanisms in males and partly via reinforcement-insensitive mechanisms in both sexes.

## Introduction

Attention deficit hyperactivity disorder (ADHD) which affects ~5% of children [1] is characterized by attention deficit, hyperactivity and impulsiveness [2], with three times higher prevalence among boys than girls [3]. The etiology of ADHD is unknown, but meta-analyses suggest a strong genetic component based on hereditary estimates in twin-studies of ~76% [4]. Although high, the concordance of monozygotic twins is not perfect, suggesting that environmental factors may act in additive or interactive ways with the genetic influence [5,6].

The cognitive dysfunction in ADHD may be affected by a lack of omega-3 (n-3) polyunsaturated fatty acids (PUFAs) during embryonic development and early life [7-9]. Although humans are able to synthesize the omega-6 (n-6) fatty acid (FA) arachidonic acid (AA) from linoleic acid (LA), and the n-3 FAs like eicosapentaenoic acid (EPA) and docosahexaenoic acid (DHA) from alpha-linolenic acid (ALA), these processes are slow and ineffective in most mammals [10], and they are also influenced by different factors like sex hormones, n-6/n-3 intake ratio and saturated fatty acid intake. Specifically, the capacity of males to transform ALA to DHA appears to be very low, and dietary access to DHA may thus perhaps be particularly important for males [11,12]. From the nutritional and anthropological point of view, humans have developed on a diet with a fair amount of n-3 FAs, whereas many industrial diets contain little of these fatty acids [13].

A lack of n-3 PUFAs in the diet of children and, conversely, feeding of n-3 PUFA to pregnant women and mothers may influence cognitive abilities in the offspring [14-20], and potentially affect the prevalence of ADHD [21-23]. Experimental studies have suggested that dietary supplementation with n-3 PUFAs may enhance both synaptic development and function [9,24,25]. Different fatty acids in neurons and glia cells might change the properties of surface and intracellular membrane compartments, membrane-associated proteins, gene transcription, neurotransmitter metabolism and activities of synaptic vesicles and transporters [13,26,27]. Previous work has also indicated a possible link between ADHD and disturbances in dopaminergic function [28-32], with downstream connections to serotonergic and glutamatergic transmissions [33,34].

In the present study we employed the spontaneously hypertensive rat (SHR), a well validated genetically determined ADHD model [35-37], to examine whether increased access to n-3 PUFAs during pregnancy and development may modulate behavioural symptoms and brain neurotransmitter metabolism [38]. Pregnant SHR dams and their offspring were fed extra n-3 PUFAs, followed by behavioural testing of the offspring in standard operant chambers to monitor reinforcement-controlled levels of activity, impulsivity and attention [39]. Moreover, video-recordings of the animals during operant testing were used to assess general locomotion. Because activity-related behaviours in rodents may be influenced via neurotransmitter interactions between dopamine (DA), serotonin (5-HT) and glutamate (Glu) located in neostriatal synapses [33,34], we subsequently measured the levels of these transmitters and their metabolites in neostriatal extracts from the very same SHR animals which had been studied behaviourally. Our results indicate that n-3 PUFA feeding may ameliorate reinforcement- and monoamine-dependent ADHD symptoms in a sex-specific manner, whereas other symptoms, independent of reinforcement and possibly correlated to changes in amino acid transmitters, were ameliorated to the same extent in both sexes.

## Methods

### Animal models

The study was approved by the Norwegian Animal Research Authority, and conducted in accordance with the laws and regulations controlling experiments on live animals in Norway. Spontaneously Hypertensive Rodents (SHR/NCrl) purchased from Charles River, Sulzfeld, Germany, were used as breeding subjects. During the first three weeks of life, the rats were under veterinarian care at the Norwegian Defence Research Establishment (Kjeller, Norway). Dams were caged singly under standard conditions (temperature ~22°C, humidity ~55%, 12 h light/dark cycle), with free access to water. The veterinarian administered feeding of either n-3 PUFA-enriched diet or chow to dams before breeding, under pregnancy and after birth. At postnatal day (PND) 25, 36 rats were shipped to the University of Oslo for behavioral testing. Thereafter, offspring of both sexes were fed similar diets as their parents, until sacrifice at PND 55–60. The weight was measured continously on SHR-rats from each experimantal group troughout the study. In order to obtain the growth rate, body weight was measured continuously on SHR-rats from each experimental group throughout the study. In the males, the n-3 PUFA diet increased the growth rate by 7.7% compared to the control-diet (with p = 0.01 and n = 8 and n= 11 respectively). Whereas n-3 PUFA diet had no effect on the female growth rate (p = 0.1, *control diet*; *n* = *10 and n*-*3 PUFA diet*; *n* = *7*).

### Experimental diet

The experimental group was given a semi-synthetic n-3 enriched feed, as described in Rokling-Andersen et al. 2009 [40]. This feed was high on the essential n-3 PUFAs EPA and DHA with 9.1% (w/w) lard and 10.4% Triomar (EPAX5500) delivered by Pronova Biocare, Lysaker, Norway. Triomar contained 55% of total n-3 FA as TAG including EPA, 300 mg/g; DHA, 190 mg/g; total n-3 FA, 580 mg/g (made up of EPA, DHA, ALA, stearidonic acid, eicosatetraenoic acid, heneicosapentaenoic acid, and docosapentaenoic acid). This dose represents about 3.6% of total energy intake of the rats and is comparable to traditional Inuit intake of marine FA [15]. In addition, 1.5% of soybean oil (Mills Soyaolje; Denofa Lilleborg, Fredrikstad, Norway) was provided to avoid essential n-6 FA deficiency.

The dietary composition (g/100 g food) was: total fat, 21; sucrose, 20; starch, 31.5; protein, 20; cellulose, 1; vitamin mixture, 1.5 and salt mixture, 5. Vitamin (Cat No: 904654) and salt mixture (Cat No: USP XVII) was bought from MP Biochemicals LLC. The feed was kept at −20 °C and given to the rats in portions sufficient for 1 day supply.

### Control-feed

The control SHRs were given lab chow (RM3 (E) from Special Diet Services, Witham, Essex CM8 3 AD, UK, http://www.sdsdiets.com/pdfs/RM3-E-FG.pdf). This feed is low on EPA and DHA, and has a n-6 to n-3 ratio of about 7:1. The fatty acid composition of this feed (w/w) were as follows: saturated fatty acids: lauric acid, 0.5 mg/g; myristic acid, 2 mg/g; palmitic acid, 3.6 mg/g; stearic acid, 0.9 mg/g and monounsaturated fatty acids: myristoleic acid, 0.1 mg/g; palmitoleic acid, 1.3 mg/g; oleic acid, 10.3 mg/g and PUFAs: LA, 11.5 mg/g; ALA, 1.7 mg/g; AA, 2.2 mg/g; docosapentaenoic acid, 0.4 mg/g. The dietary composition (g/100 g food) was: total fat, 4.2; sucrose, 5.7; starch, 33.9; protein, 22.4; cellulose, 3.9; hemicellulose, 9.2; dietary fibre, 15.4; vitamin mixture, 0.5 and salt mixture, 4.6. The diet was kept at −20 °C until fed to the animals.

### Behavioural analyses

At PND 21 the offsprings were moved to a separate location for behavioural testing. All 41 sessions included habituation, training, shaping and testing with reinforcers. The duration and reinforcement scedule of the different sessions is described in the summary of the experimental procedure (table 1). Sessions used in the analyses lasted for 90 min.

**Table 1:** The reinforcement was given using either a fixed time schedule of reinforcement (FT), continuous reinforcement schedule (CRF) or a variable interval schedule of reinforcement (VI)

**Behavioral procedure**	**Session number**	**Reinforcement schedule**
Habituation, 30 min	1	
Magazine training, 30 min	2 – 3	FT 10 seconds
Flap training	4 – 5	CRF
Shaping of lever-pressing	6 – 7	
30 minutes session	8 – 13	VI 3 seconds
90 minutes session, 16 chambers	14 – 41	VI 180 seconds

A total of 36 animals were tested using a two-lever visual discrimination task [39] with one session each day for 34 days (the last 28 sessions were used in the analyses). The animals were also video-recorded during the operant task in sessions 13, 24, and 34 to monitor spontaneous locomotion, and the results were averaged for each rat. The n-3 PUFA-fed group included 8 males and 7 females, whereas 11 males and 10 females served as control-fed reference animals. Testing of the experimental and control groups took place at different time points. During habituation and response acquisition, two offsprings were housed in one transparent cage 41 × 25 × 25 cm (height), whereas during response acquisition and throughout the rest of the study, the animals were housed individually in the same type of cages. The rats had free access to water during the habituation sessions, following which they were deprived of water for 21 h a day throughout the rest of the study. The behavioural testing took place between 0900 and 1400 h.

Table 1: Summary of the behavioural procedures and reinforcement schedule.

#### Apparatus

The animals were tested in 16 operant chambers (Campden Instruments) enclosed in sound-resistant outer housings [39,41]. The chambers were ventilated and equipped with a grid floor, and the animals’ working space was 25 × 25 × 30 (height) cm. The chambers were equipped with two retractable levers requiring a dead weight of at least 3 g to activate a micro-switch. A 2.8 W cue light was located above each lever. The reinforcer (0.05 mL tap water) was delivered by a liquid dipper located in a small recessed cubicle, where a 2.8 W cue light lit up. A 7 × 5 cm transparent plastic top-hinged flap separated the cubicle from the animal’s working space. The computer program LabVIEW 7.1 (National Instruments *LabVIEW*, Austin, Texas, USA, 2004) recorded the behaviour and scheduled reinforcements and lights. Each operant chamber was equipped with a video camera (Mini Color Hidden Cameras (420TVL, 0,1lux) from Tracer Technology Co. Ltd, Taiwan) positioned to capture the entire working space, in the upper rear corner of the ceiling at an angle of 45 ^o^. The DVR Live Capture computer program (Novus Security, 2009, Warsaw, Poland) controlled the cameras and saved the video files for analyses.

#### Operant testing

Prior to behavioural testing, the rats were semi-randomly assigned an operant chamber. Following habituation in the operant chamber, the animals were trained to lever-press and then run for additional sessions to strengthen the newly learned behaviour. In the operant task, two levers were used. Pressing the lever, which was signalled by a lit cue light located above the lever, produced reinforcements according to a variable interval 180 s schedules of reinforcement (VI 180 s). During this period, the cue light above the alternative lever was off, and lever press had no consequences. During reinforcer delivery, the cue light above the lever was turned off, and a 2.8 W cue light was lit in the water cubicle. The lever producing reinforcers alternated unpredictably between the two lever-alternatives, but stayed the same until a reinforcer was produced by a lever-press. Following reinforcer delivery, the computer program semi-randomly selected which lever would produce the next reinforcer. To avoid development of lever-preferences, the program allowed a maximum of four consecutive reinforcers on the same lever.

#### Measure of reinforcement-controlled behaviour

Recording was made of number of presses on the reinforcer-producing lever and on the alternative lever, number of reinforcers produced and collected, and the time of the events. Percentage of responses on the lever producing reinforcers (for all responses, and for the first response following reinforcer delivery), and the time between two responses (inter-response time, IRT), were calculated. Attention was operationalized as the percentage of responses on that lever which produced reinforcers (the animal had to pay attention to and press the lever signalled by the lit cue light above the lever, i.e. stimulus control). Hyperactivity was operationalized as the total number of lever-presses on the two levers combined. The IRTs were split into responses with IRTs longer than 0.67 s and shorter than 0.67 s. Number of responses with short IRTs (< 0.67 s) was used as a measure of impulsivity (“premature responding” or “inability to wait”).

#### Video recordings

The animals were video-recorded during the operant task in 3 sessions, which were chosen to represent the spontaneous activity early, in the middle of and late in the experimental testing period. The cameras recorded 15 frames per second, and frame-to-frame analyses of changes in pixels, which occurred whenever the animal moved, were performed by using the computer program Musical Gestures Toolbox for audio and video analysis [42]. The total number of pixel-changes was used to quantitate movements. Each of the 3 sessions was divided into 5 segments in order to analyse within-session changes in locomotion in the experimental and control groups. To reduce noise, pixel-changes were averaged across 15 frames and a noise reduction threshold (0.25) and filter (8) were used.

#### Analysis of behavioural data

All statistical analyses were performed in Statistica 6.0 (StatSoft. *Statistica for Windows*, StatSoft Inc., Tulsa, OK, 2005). Data were evaluated either by multivariate analyses using Wilks lambda (MANOVAs) when the degrees of freedom relative to the number of levels of the repeated factor permitted this approach, or by univariate analyses of variance (ANOVAs), adjusting the degrees of freedom with the Huynh–Feldt epsilon [43]. Sessions were used as the within-individual factor in the operant task, whereas sessions and segments were used as the within-individual factor in the video analyses. n-3 PUFA-feeding was used as the between-individuals factor in both analyses. Session were included as a factor to look for changes in behaviour across development. Post-hoc tests on main effects were performed using the Unequal N HSD test. Preparation of graphs was performed using Prism (GraphPad Software Inc.) and the graphs represent means ± SEM for each group (n = 7–11).

### Biochemical analyses

#### Chemicals

Monoamine and amino acid analyses were done with high performance liquid chromatography (HPLC). L-amino acid standards, including aspartic acid (Asp), glutamic acid (Glu), serine (Ser), glutamine (Gln) and glycine (Gly) were obtained from Pierce (Rockford, Ill., USA), whereas taurine (Tau), γ-amino butyric acid (GABA), and α-amino adipic acid were from Sigma (Sigma-Aldrich, Steinheim, Germany). The monoamine standards dopamine (DA), homovanillic acid (HVA), serotonin (5-HT), 5-hydroxyindole-3-acetic acid (HIAA) and 3,4-hydroxybenzylamine (DHBA), as well as HClO_4_ and ascorbic acid were also obtained from Sigma. The BCA-assay kit (Thermo-Scientific, Rockford, USA), and n-hexane (Merck, Darmstadt, Germany) were bought from VWR. Solutions were made with purified distilled water (Milli-Q Advantage A10, Millipore).

#### Sample collection

Following behavioural testing, the SHR rats as well as age-matched Wistar Kyoto rats (WKY/NHsd) [37] were stunned and rapidly decapitated at PND 55–60 (the WKY/NHsd were used as additional reference strain in the biochemical analyses). The neostriata were removed, frozen in liquid N_2,_ and stored at −70°C until sample preparation and analyses.

#### Extract preparation and protein assay

The tissues were rapidly weighed and homogenized by hand with 20 strokes in 500 μL ice cold 0.2 M HClO_4,_ using a glass/teflon Potter-Elvehjem homogenizer. This suspension was mixed with an equal volume of DHBA (used as internal standard for monoamines) in 0.12 mM ascorbic acid to a final concentration of 0.227 μM DHBA. The homogenates were centrifuged for 20 min at 15000 × g at 2 °C in a Sorvall RMC-14-microcentrifuge. The pellets were frozen at −40 °C for later protein determination, performed by dissolving pellets in 0.1 M NaOH and measuring protein content by the BCA assay [44]. The supernatants were extracted with equal volumes of n-hexane to reduce lipid contamination, and the top layer was discharged. For amino acid analyses, parts of the delipidized HClO_4_ extracts were mixed with a solution of α-amino adipic acid (used as internal standard) to a final concentration of 10 μM in a total volume of 1.9 mL. These extracts were neutralized to pH 7.2 with ice cold KOH and centrifuged for 20 min at 15000 × g at 2 °C as described above. The supernatants were stored at −70 °C until analyses. The samples were then transferred into glass-vials by filtering through Nylon-66 micro filters (0.22 μm) from Nalgene (Rochester, New York, USA), before HPLC analysis.

#### Monoamine analysis

**Figure 1:**
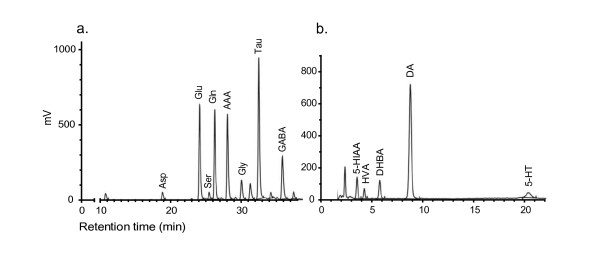
**Chromatographic profiles of rat neostriatal extracts.** (**a**) Amino acids in a WKY control-fed female, and (**b**) monoamines in a control-fed SHR male. Panel **a**) shows the peaks of aspartic acid (asp), glutamate (Glu), serine (Ser), glutamine (Gln), α-amino adipic acid (AAA), glycine (Gly), taurine (Tau) and γ-amino butyric acid (GABA). Panel **b**) shows the peaks of 5-hydroxyindol-3-acetic acid (5-HIAA), homovanillic acid (HVA), 3,4-hydroxybenzylamine (DHBA), dopamine (DA) and serotonin (5-HT).

Column and mobile phases were selected for analysis of catecholamine content in plasma and was supplied by Chromsystems (Germany). The frozen extracts were used directly for analyses of total DA and 5-HT, as well as their metabolites HVA and 5-HIAA, using a reversed-phase HPLC (Shimadzu, Kyoto, Japan) with electrochemical detection (ECD; Decade II with Sencell flow electrode, Antec Leyden) set to a working potential of 0.6 V. Each sample was eluted for 40 min with a flow rate at 1.3 mL/min and a representative chromatogram is shown in Figure 1b. External standard solutions of DA, HVA, 5-HT, 5-HIAA and DHBA were analysed on the same day. The chromatograms were analysed using the software Lab Solutions (Shimadzu). Monoamine concentrations are expressed in pmol/mg total neostriatal protein.

#### Amino acid analysis

Using a Chromspher 5 C18 column of 25 cm length and 4.6 mm inner diameter (Varian), total amino acids in the neostriatum extracts were analysed [45], using reversed-phase HPLC fitted with a fluorescence detector (Shimadzu, Kyoto, Japan) after derivatization with o-phthaldialdehyde (OPA; Sigma). The mobile phase comprised 75% 50 mM phosphate buffer, pH 5.25, and 25% methanol (v/v), changing linearly to 25% phosphate buffer and 75% methanol during 26.5 min, after which the methanol concentration was linearly reduced to 15%. Each sample was eluted for 45 min with a flow rate at 0.4 mL/min and a representative chromatogram is shown in Figure 1a. A mixture of the amino acids of interest was used as external standards in a concentration of 100 μM. The chromatograms were analysed using the software Lab Solutions (Shimadzu). Amino acid concentrations are expressed in nmol/mg total neostriatal protein.

#### Statistical analysis

Statistical significance of biochemical differences between samples was determined by unpaired, two-tailed Student’s *t*-test, where p < 0.05 is defined as significant. Level of significance is symbolized with * (p-value ≤ 0.05), ** (p-value ≤ 0.01 and *** (p-value ≤ 0.001). Statistical analyses as well as preparation of graphs were performed using Prism (GraphPad Software Inc), and the graphs display the means ± SEM for each group (n = 4–8).

## Results

### Behavioural analysis

#### Operant testing

**Figure 2:**
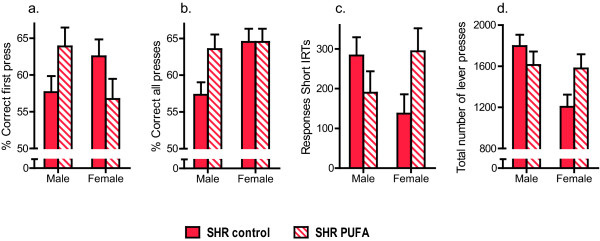
**Analysis of reinforcement-controlled behaviour.** (**a**) Attention (stimulus control; percentage of responses on the reinforcer-producing lever) for the first lever-press following reinforcer delivery (sex × supplementation, F1,32 = 5.99, p = 0.02); (**b**) Attention (stimulus control; percentage of responses on the reinforcer-producing lever) for all responses (sex × supplementation, F1,32 = 3.66, p = 0.06); (**c**) Impulsivity (‘premature response’, lever presses with inter-response times <0.67 s) (sex × supplementation, F1,32 = 5.81 p = 0.02); (**d**) Lever-directed hyperactivity (all lever-presses) (sex × supplementation, F1,32 = 4.94, p = 0.03). Data are presented as means ± SEM (n = 7–11).

Overall, n-3 PUFA supplementation interacted with sex in the SHR animals, improving percentage of presses on the lever producing reinforcers (stimulus control) in n-3 PUFA-fed males, but having the opposite effect in females (Figure 2a- 2b). Analyses of stimulus control for all lever presses showed a statistically significant main effect of session (*F*(*2*,*31*) = *11*.*31*; *p* < *0*.*01*). Trends were observed for a main effect of sex (*F*(*1*,*32*) = *3*.*45*; *p* = *0*.*07*), and for a sex × feed condition interaction (*F*(*1*,*32*) = *3*.*66*; *p* = *0*.*06*). Analyses of stimulus control for the first lever press following reinforcer delivery (Figure 2a) also showed a statistically significant sex × feed condition interaction effect (*F*(*1*,*32*) = *5*.*99*; *p* = *0*.*02*). No significant effects were found in the Unequal N HSD post-hoc analyses of the significant sex × food condition interaction effect.

No statistically significant effects were observed during analyses of number of reinforcers collected. Out of the possible 30 reinforcers per session, the mean number of reinforcers collected was similar in all groups across sessions, and ranged from 28.3 (n-3 PUFA-treated males, session 27) to 29 (control-fed male SHRs, session 6).

Number of responses with short IRTs was decreased in n-3 PUFAs supplemented males but increased in n-3 PUFA supplemented females (Figure 2c). The analyses showed a significant main effect of session (*F*(*2*,*31*) = *4*.*23*; *p* = *0*.*02*) and a significant sex × feed condition interaction (*F*(*1*,*32*) = *5*.*81*; *p* = *0*.*02*). Also, a significant sex × feed condition × session interaction was found (*F*(*2*,*31*) = *5*.*60*; *p* < *0*.*01*). No significant effects were observed in the post-hoc analyses of the significant sex × feed condition interaction effect using the Unequal N HSD test.

Supplementation with n-3 PUFAs reduced lever-directed hyperactivity in males and increased hyperactivity in females (Figure 2d). The analyses showed significant main effects of sex (*F*(*1*,*32*) = *6*.*23*; *p* = *0*.*02*), session (*F*(*2*,*31*) = *8*.*62*; *p* < *0*.*01*) and a significant sex × feed condition interaction (*F*(*1*,*32*) = *4*.*94*; *p* = *0*.*03*). Unequal N post-hoc analyses of the significant sex × food condition interaction showed that control-fed SHR males emitted significantly more lever-presses than control-fed female SHRs (*p* = *0*.*01*).

#### Locomotor testing

**Figure 3:**
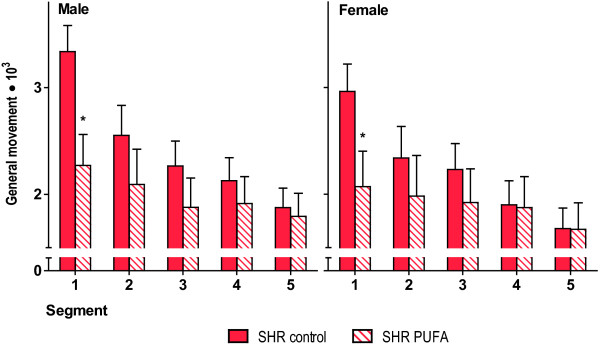
**Analysis of general locomotion.** Video analyses of pixel-changes showed that n-3 PUFA supplementation reduced locomotion in the initial 18 min segments of the 90 min sessions in both males and females. For the first segment, comparison of general movement for PUFA-supplemented SHR vs. SHR control is significantly different, as symbolized by * (p ≤ 0.05), using unpaired two-tailed Student’s *t*-test. Data are presented as means ± SEM (n = 7–11).

Analyses of video data showed that n-3 PUFA-supplemented rats had reduced locomotion in the initial segments of the sessions in both males and females (Figure 3). The analyses showed significant main effects of session (*F*(*2*,*30*) = *8*.*63*; *p* < *0*.*01*) and segment (*F*(*4*,*28*) = *14*.*77*; *p* < *0*.*001*,) as well as significant sex × segment (*F*(*4*,*28*) = *3*.*29*; *p* < *0*.*03*), feed condition × segment (*F*(*4*,*28*) = *4*.*63*; *p* < *0*.*01*) and session × segment (*F* (*8*,*24*) = *6*.*31*; *p* < *0*.*001*) interaction. In addition, the analyses showed a significant sex × feed condition × session interaction (*F*(*2*,*30*) = *3*.*54*; *p* = *0*.*04*) and a significant food condition × session × segment interaction (*F*(*8*,*24*) = *2*.*44*; *p* = *0*.*04*). This means that all the SHRs increased their general activity between early and middle session, and within sessions there was a decrease in general activity across segments. The males showed more general activity across segments relative to female SHRs. Comparison of general movement for PUFA-supplemented SHR vs. SHR control showed that general movement was significantly different for the first segment, using unpaired two-tailed Student’s *t*-test.

#### Behavioural summary

Taken together, n-3 PUFA supplementation significantly enhanced reinforcement-controlled attention (Figure 2a-b) and reduced lever-directed hyperactivity (Figure 2d) and impulsivity in SHR males (Figure 2c), whereas the opposite or no effects were observed in females (Figure 2c-d). In contrast, general locomotion was reduced by n-3 PUFA-feeding to a similar extent in both sexes (Figure 3).

### Biochemical analyses

#### Monoamine transmitters

**Figure 4:**
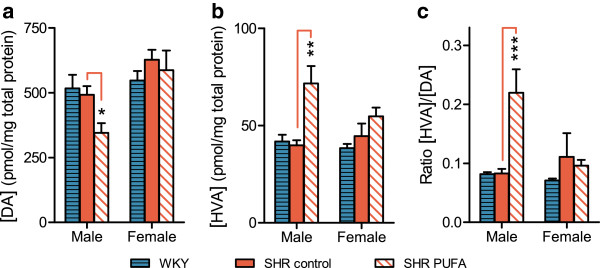
**Dopamine (DA) and homovanillic acid (HVA) levels in neostriatum.** n-3 PUFA supplementation modulated DA turnover in male SHR: (**a**) DA and (**b**) HVA levels were used to determine (**c**) HVA/DA ratios. Data are presented as means ± SEM (n = 4–8) and the level of significance is symbolized by * (p-value ≤ 0.05), ** (p-value ≤ 0.01) or *** (p-value ≤ 0.001). The p-values are calculated by comparison with the mid bar representing control-fed SHRs. The analyses were performed by HPLC using electrochemical detection.

Dietary supplementation with n-3 PUFA to the dams and offspring changed DA and 5-HT dynamics in the SHR neostriata in a sex-specific manner, with significant effects in the male SHR offspring only. DA levels were reduced by 30 ± 11% in the n-3 PUFA-fed male SHRs (n = 4) as compared to control-fed male SHRs (n = 8), p = 0.03. In all other animals, DA levels remained similar to the WKY controls (Figure 4a). In the neostriatum, DA is exclusively present in the terminals of the nigrostriatal fibers, with DA levels representing a balance between DA release, DA reuptake/synthesis and DA degradation. Thus, we compared levels of DA with the DA degradation product HVA, a measure indicating biochemical DA utilization in dopaminergic synapses [46,47]. Males fed n-3 PUFA showed a 80 ± 18% increase in HVA levels (n = 4) in neostriatum (Figure 4b), leading to a HVA/DA ratio of 0.22 ± 0.04 in the male SHR, which was more than twice that obtained in the female SHR and the control-fed SHR and WKY (Figure 4c).

**Figure 5:**
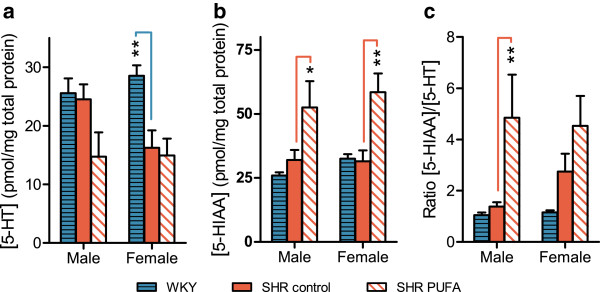
**Serotonin (5-HT) and 5-hydroxyindole-3-acetic acid (5-HIAA) levels in neostriatum.** n-3 PUFA supplementation modulates 5-HT turnover in both male and female SHR rats. Levels of (**a**) 5-HT and (**b**) 5-HIAA were used to determine (**c**) 5-HIAA/5-HT ratios. The data are presented as means ± SEM (n = 4–8) and the level of significance is symbolized with * (p-value ≤ 0.05), ** (p-value ≤ 0.01) or *** (p-value ≤ 0.001). The p-values are calculated by comparison with the mid bar, which represents control-fed SHRs. The analyses were performed by HPLC using electrochemical detection.

Similar to the DA system, 5-HT in the neostriatum is exclusively present in nerve terminals [28]. In the WKY controls, total 5-HT levels were similar in both sexes, whereas in the SHRs, they were slightly higher in control-fed males than females. This sex difference was, however, obliterated by n-3 PUFA supplementation, with 5-HT levels decreasing by 40 ± 18% in the males (n = 4) as compared to control-fed SHR (n = 8, p = 0.06), whereas the female 5-HT levels remained unchanged after n-3 PUFA supplementation. At the same time, n-3 PUFA feeding enhanced the levels of the degradation product 5-HIAA [28] in both sexes (Figure 5b), with the 5-HIAA/5-HT ratios indicating 5-HT turnover rates of 4.8 ± 1.7 (n = 4) and 4.5 ± 1.2 (n = 4) in the male and female n-3 PUFA-fed SHRs, respectively. In contrast, control-fed male and female SHRs had ratios of 1.3 ± 0.4 and 3 ± 1.1, respectively, whereas WKY controls of both sexes showed ratios of 1.1 ± 0.1 (n = 5) and 1.2 ± 0.1 (n = 5), respectively (Figure 5c). Thus, PUFA-feeding enhanced 5-HT utilization in the SHRs to the same levels in both sexes. Interestingly, the statistically significant effect was restricted to the males, whereas the female SHR showed a partial increase already without PUFA supplements.

#### Amino acid transmitters

**Figure 6:**
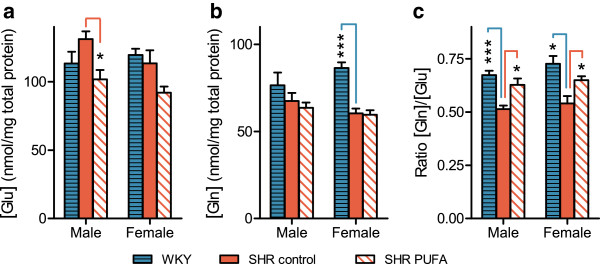
**Glutamate (Glu) and glutamine (Gln) levels in neostriatum.** n-3 PUFA supplementation modulates Gln/Glu turnover in both male and female SHR rats. Levels of (**a**) Glu and (**b**) Gln were used to determine (**c**) Gln/Glu ratios. Control-fed SHRs of both sexes showed significant ratio reductions when compared to WKY controls, and n-3 PUFA feeding reversed these ratios in both SHR sexes. Data are presented as means ± SEM (n = 4–8) and the level of significance is marked with * (p-value ≤ 0.05), ** (p-value ≤ 0.01) or *** (p-value ≤ 0.001). The p-values are calculated by comparison with the mid bar representing control SHRs.

Supplementation with n-3 PUFA also modulated amino acid transmitters in the neostriata. Recent studies suggest that Gln/Glu ratios may indicate the efficacy of presynaptic glutamatergic signalling *in vivo*[48,49], In our study, levels of the major excitatory transmitter Glu were reduced in n-3 PUFA-fed SHRs of both sexes, with males showing a significant reduction of 22% (n = 4, p = 0.02). And a similar effect occurring in the females (Figure 6a). In contrast, levels of the precursor/metabolite amino acid Gln remained stable in SHRs of both sexes (Figure 6b), leading to significantly higher Gln/Glu ratios in both sexes of n-3 PUFA-fed SHRs when compared to control-fed SHRs. Specifically, control SHRs of both sexes had a Gln/Glu-ratio of 0.5, whereas n-3 PUFA-fed SHRs of both sexes showed a significant 21% increase of Gln/Glu ratios (p = 4.9 × 10^-4^). These latter results approached those observed in the WKY controls, where both sexes showed a 33% higher Gln/Glu ratio than that observed in the control-fed SHRs (p = 3.2 × 10^-4^), with a mean value of 0.7. Hence, n-3 PUFA-feeding appeared to reverse the abnormally low Gln/Glu ratio in the control-fed SHRs towards control WKY-values in both sexes of the SHR animals (Figure 6c). Thus, our data suggest that control-fed SHR animals had a subnormal glutamatergic activity, and that this was partly normalized in the n-3 PUFA-supplemented animals.

**Figure 7:**
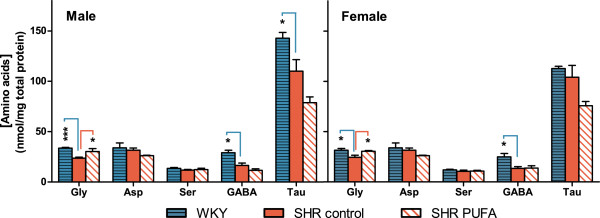
**Concentrations of amino acids in neostriatal extracts.** Both male and female control-fed SHRs showed significantly reduced levels of glycine (Gly) when compared to WKY controls, and n-3 PUFA supplementation increased Gly levels significantly in both SHR sexes. In contrast, GABA levels from SHR controls were significantly lower than in WKY controls of both sexes, and n-3 PUFA supplementation did not change the level of GABA. Taurine (Tau) levels were significantly lower in the male SHR controls than in the male WKY controls. n-3 PUFA supplementation promoted a small, non-significant decrease in Tau levels in both sexes. Aspartate (Asp) and serine (Ser) remained unchanged. Data are presented as means ± SEM (n = 4–8), and the level of significance is symbolized with * (p-value ≤ 0.05), ** (p-value ≤ 0.01) or *** (p-value ≤ 0.001). The p-values are calculated by comparison with the mid bar representing control-fed SHRs.

In addition to these changes in the major neurotransmitter monoamines and amino acids, minor effects were also seen in glycine (Gly). This bifunctional amino acid, which is active both as an inhibitory transmitter in the brain stem and spinal cord, and as an obligatory co-ligand of the NMDA-type of Glu receptor in excitatory synapses [50,51], was significantly increased after n-3 PUFA-supplementation in both SHR sexes. When compared to the male and female WKY controls, neostriatal Gly levels were significantly lower in control-fed SHRs of both sexes (by 42 and 28%, p = 2.4 × 10^-4^ and p= 0.04), respectively (Figure 7). Following n-3 PUFA supplementation, Gly levels were changed to a level similar to what was seen in WKY neostriata, by 24 and 28% increases (p = 0.03 and p = 0.05), respectively. Thus, our observations imply both a genetic difference between the Gly systems in the SHR and WKY strains and an effect of n-3 PUFAs on the metabolism of neostriatal Gly in the SHRs.

In contrast to the glycinergic system, n-3 PUFA feeding had no significant effect on GABA levels in the SHR of either sex. Hence, the diet-induced major changes in excitatory glutamatergic fibers as well as the modulatory dopaminergic and serotoninergic synaptic inputs to the neostriatal cells seen in the SHR animals (see above), did not lead to major changes in the local GABAergic transmission present in neostriatum.

Finally, the levels of Asp and Ser were unchanged by dietary n-3 PUFAs in both male and female SHRs. Moreover, although the concentration of taurine (Tau) appeared to respond to n-3 PUFA feeding by decreasing levels in both male and female SHRs, these changes were not significant (p = 0.08 and 0.065 respectively). A potential effect of Tau in modulating DA release in the ventral striatum, of importance for reinforcement mechanisms [52] and possibly ADHD in the SHR, therefore remains uncertain. These data also support the idea that n-3 PUFA supplementation predominantly affects synaptically active amino acids and not general amino acid metabolism.

## Discussion

In this study, dietary n-3 PUFA supplementation to SHRs was associated with two distinct types of changes in ADHD-like behaviours which occured in synchrony with changes in neurotransmitter systems. One of these included reduced levels of reinforcer-controlled activity, impulsiveness and inattention in male SHR, with no or opposite effects in the female SHRs. Neurotransmitter dynamics in neostriatum of these animals were also changed, showing enhanced DA and 5-HT turnover, which again were predominantly seen in male SHRs. In contrast, the other change included a reduction in general locomotor activity in both SHR sexes, which appeared to correlate with changes in levels of several neuroactive amino acids in both male and female SHRs. Thus, our data indicate major sex-specific effects on neurotransmitter systems in the PUFA-fed animals, with the males having unique enhancements of both DA and 5-HT in the neostriatum [53]. Although the 5-HT degradation product 5-HIAA also was significantly increased in females, with a similar turnover ratio to that seen in males, the turnover in female SHRs was not statistically significant. Even though the mechanisms behind these effects of n-3 PUFAs are unclear [9,27,54,55], our data correlate well with the behavioural observations, which also showed sex differences, with males improving on all operant measures, whereas females did not. This similarity suggests a possible causal relation between the n-3 PUFA-induced increase of modulatory neurotransmission [56] and improved behaviour [36].

Reinforcement mechanisms in the CNS are mostly mediated via DA synapses [57-59], which are heavily concentrated in neostriatal brain regions, particularly in the dorsal and ventral caudate-putamen [60]. Here, excitatory Glu inputs from cerebral cortex and thalamus predominantly terminate on efferent GABAergic cells, with the widely distributed DA and 5-HT modulatory synaptic inputs from the brain stem having the ability to modulate the efficacy of the Glu-to-GABA synapses, thereby determining to what extent efferent GABAergic information will be transmitted out of the basal ganglia [61]. This synaptic effect, mediated trough an undefined mechanism being involved in the neurobiological changes occurring in the n-3 PUFA-fed animals, may suggest enhanced action potential firing frequencies, structural synaptic changes (e.g. increased number of vesicles or synapses) or enhanced biochemical capacity, vesicle storage capacity or enhanced neurotransmitter levels [62-66].

In contrast, effects on locomotion appear to represent a distinct target, with general, non-operant locomotion activities being sensitive to n-3 PUFAs in both sexes. Given that the locomotion occurred continuously during the operant test, and that frame-to-frame analyses of video-recordings detected changes in pixels whenever the animals moved, it is clear that this measure reflected both operant behaviour controlled by the scheduled reinforcers as well as general movements. The interpretation that operant behaviour and video-measured locomotion represent two distinct phenomena is also supported by the dissociation of reinforcement-controlled lever pressing and changes in the video-measured locomotion observed in female SHRs, where n-3 PUFA feeding increased levels of operant responses but reduced initial video-measured locomotion.

Because the PUFA-induced reduction in non-operant behaviour locomotor activity occurred in both sexes, it is of great interest that those neuroactive transmitter amino acids which responded to n-3 PUFAs also showed similar patterns in the two sexes. Specifically, these included reduced plasma levels of Glu and enhanced levels of Gly, with no changes in Gln and GABA. Because the Gln/Glu ratio was significantly increased, glutamatergic neurotransmission in neostriatum of the SHRs may be enhanced by dietary n-3 PUFA supplementation in both sexes. Previous analysis has suggested that motor activity may be modulated by Glu receptor activation [33], in agreement with our observations.

The levels of Gly showed similar differences as those found in the Gln/Glu ratios; with low levels in the control-fed SHRs, and PUFA-induced increase in levels seen in both SHR sexes. Although Gly receptors are present in the rat striatum, and are proposed to modulate both cholinergic, dopaminergic and glutamatergic transmission [67-69], the importance of increased Gly levels in the striata of n-3 PUFA supplemented SHRs remains unknown [70-73]. Previous work has also indicated changes in Tau levels in male SHRs when compared to WKY controls [74], suggesting a possible role for Tau in association with ADHD. Indeed, Tau changes may lead to impaired regulation of Gly, GABA and/or DA signalling [52,75-78]. However, Tau levels were only insignificantly reduced following n-3 PUFA feeding, making it unlikely that these changes represent an important mechanism for mediating n-3 PUFA-induced amelioration.

Finally, the PUFA-induced normalization of the Gln/Glu ratios as well as Gly levels, which occur in both female and male SHRs, is of considerable interest. This contrasts to the major PUFA-induced changes found in the DA and 5-HT turnover, seen predominantly in the male SHRs. Intuitively, these dietary effects of n-3 PUFA in ADHD models like SHR would be expected to normalize both groups of neurotransmitters. However, given the well-known distinct functional organizations of the quantitatively predominant amino acidergic synapses in the neostriatum as compared to the minor but widespread modulatory monoaminergic synaptic systems [79], our data appear consistent with the interpretation that n-3 PUFA feeding induced strong activation of the modulatory DA and 5-HT systems in order to normalize the functionally more important amino acidergic neurotransmitter systems. It should also be emphasized that our study only represents a small number of possible factors involved in ADHD-development. Moreover, other studies have also observed sex differences in other important neurochemical parameters like the dopamine D1 and D2 receptors [80], or monoamine-catabolizing enzymes like COMT [81,82]. Finally, a potential weakness of our study is that the control group is fed considerably less fat than the n-3 PUFA-fed group. However, recent studies [83] demonstrated that adult male Long Evans rats given a high-fat diet developed a decrease, not an increase in their DA turnover in the ventral striatum, making it highly unlikely that the high-fat diet *per se* employed in this study is responsible for the enhancement of DA turnover which occures in the male SHRs.

## Conclusion

Our study indicates that dietary supplementation of n-3 PUFA to SHR dams and their offspring induces two distinct sets of changes in the offsprings: The first included activation of presynaptic neostriatal DA and 5-HT signalling as well as several reinforcement mechanisms [84], all restricted to the male SHR, suggesting that this effect may preferentially improve male ADHD-like symptoms; In contrast the second included a non-reinforcement but PUFA-induced improvement in behavioural responses in both male and female ADHD animals, and changing amino acid transmitters to the same extent in both sexes. Thus, n-3 PUFA may partly ameliorate ADHD-like behaviour via reinforcer-induced systems restricted to males, and partly via reinforcer-insensitive systems present in both sexes.

## Competing interests

The authors declare that they have no competing interests.

## Authors' contributions

The study was designed by TS, CAD, EBJ and SIW. GW performed breeding and behavioural testing. KSD, BÅR and ILB did biochemical and EBJ and THS behavioural analysis. KSD, THS, ILB, EBJ, CAD and SIW wrote the manuscript. TS was deceased before finishing this final version of the manuscript. All authors except TS read and approved the final manuscript.
